# The prognostic and immunological role of MYB: from bladder cancer validation to pan-cancer analysis

**DOI:** 10.1042/BSR20222627

**Published:** 2023-04-06

**Authors:** Xiaobo Cui, Chao Zhang, Liqi Zhang, Huaqing Yan

**Affiliations:** 1 Department of Urology, Ningbo Medical Center Lihuili Hospital, Ningbo, Zhejiang 315000, P.R. China; 2 Department of Nephrology, Ningbo Medical Center Lihuili Hospital, Ningbo, Zhejiang 315000, P.R. China; 3 Department of Reproductive Medicine, The First Affiliated Hospital of Ningbo University, Ningbo, Zhejiang 315000, P.R. China

**Keywords:** bioinformatic analysis, c-myb, immunization, pan-cancer, prognosis

## Abstract

**Background:** MYB proto-oncogene is verified as a transcription factor. Although emerging evidence showed that MYB plays a critical part in tumor progression and immunity, a systematic pan-cancer analysis of MYB still remains to be performed for determining whether MYB could serve as a biomarker for cancer screening, prognosis prediction and accurate therapy design in various human cancers.

**Methods:** In the present study, we performed qRT-PCR, wound healing assay and transwell assay to validate the expression level and biological function of MYB in bladder cancer. Then, we utilized several open-source databases including UCSC Xena database, TCGA, GTEx, etc. Online tools was used to process the raw data from UCSC Xena database.

**Results:** We found that the expression level of MYB is significantly higher in bladder cancer cell lines than urothelial cells. Further experiments confirmed that overexpression of MYB enhanced the ability of migration in bladder cancer. Next, we found that the expression level of MYB is significantly higher in most cancers. Meanwhile, MYB expression was positively or negatively related with the prognosis in different cancer types. In addition, MYB expression is significantly related to immune score and immune cells in most cancer types. Moreover, MYB act as an immunotherapy biomarker superior to several traditional immunotherapy biomarkers. Finally, deep deletion was the most frequent genetic alteration of MYB.

**Conclusion:** MYB may serve as a powerful biomarker for tumor screening, prognostic, individualized treatment strategy in a broad range of malignancies.

## Introduction

Cancer remains the dominant cause of morbidity and mortality globally and lead to huge burden for public health and economics. In the United States, it is estimated that there would be 1,918,030 cancer cases newly diagnosed in 2022, which means approximately 5250 new cases would be identified each day [1]. The pathogenesis of cancer is complex and unclarified. In our previous study, changes in both molecular and macroscopic level could lead to a rise in cancer risk [2,3]. Traditional therapies mainly include surgery, radiotherapy and chemotherapy; recent years the advances in immunotherapy and molecular targeting therapy bring about great improvement in treatment for cancer. Emerging results indicate that immunotherapy showed efficiency in various cancers and in specific cancer type it reaches a surprisingly 100% complete response rate [4]. However, the resistance to immune checkpoint blockade is still a major problem for immunotherapy which fueled a wave of research into the characters of tumor cells and tumor immune microenvironment [5,6].

Tumor cells and tumor immune microenvironment (TIME) are closely related and affect each other. Tumor cells gradually escape from immune surveillance by shaping an immunosuppressive TIME in its progression and an immunosuppressive TIME promotes tumor growth by various mechanisms including the depletion of tumor-infiltrating T cells, the inhibitory role of immune checkpoint genes such as VISTA, TIM-3 and LAG-3 and inhibitory immune cells like Tregs, TAMs and MDSCs [7]. Therefore, understanding TIME is equally critical with the study of tumor cells in order to achieve better cancer treatment strategies. Recently, Carlson et al. found that MYB orchestrates T-cell exhaustion and response to checkpoint inhibition during chronic infection [8]. MYB is vital for the development of CD62L+ T_PEX_ cells, a cluster of cells identified from the precursors of exhausted T cells via single-cell RNA sequencing. These cells have superior self-renewal, multipotency and long-term proliferative capacity but loss of MYB impairs the differentiation of these cells and finally destroy the complete CD8+ T-cell response. Moreover, MYB accounts for the functional exhaustion of CD8+ T cells during chronic infection. Thus, MYB related two key properties of exhausted CD8+ T cells: no function and longevity.

MYB proto-oncogene, transcription factor (MYB), as a protein coding gene, is located on chromosome 6q23.3 with 20 exons. As a main member of MYB gene family which includes MYB, MYBL1 and MYBL2, MYB is involved in the control of cell survival, proliferation and differentiation and, as a transcription factor, serves as a convergence of numerous signaling pathways, which is essential for tumor progression [9]. In leukemia, mebendazole modulates MYB degradation by interfering with the heat shock protein 70 and exhibit the ability of impairing acute myeloid leukemia (AML) growth *in vivo* involving SP70/HSC70 chaperone pathway. It is reported that momentary exposure with mebendazole is enough to inhibit the colony formation ability of AML cells, and further study confirmed mebendazole availability for impairing AML progression in mouse xenotransplantation experiments. Meanwhile, this exposure did not impair normal cord blood-derived cells, indicating that MYB represents a safe and novel therapeutic approach for AML [10]. In breast cancer, MYB regulates the response of DNA damage, suggesting a potential therapeutic target in ER+ breast cancer and possibly other cancer types via DNA damage response/repair pathways [11]. Meanwhile, the oncogenic role of MYB is identified in esophageal adenocarcinoma, ovarian cancer, salivary adenoid cystic carcinoma, pancreatic tumor, etc. [12–16].

Apart from the critical role of MYB in cancer as an oncogene, MYB also affects the immune microenvironment. Gautam et al. reported that MYB promoted curative antitumor immunity and regulated CD8+ T-cell stemness [17]. Cicirò et al. reviewed studies about MYB family members and identified its role as a potential therapeutic target [18]. However, the prognostic value and immunity of MYB in various human cancers is not clearly elucidated. Thus, a systematic pan-cancer analysis of MYB still remains urgently needed to determine whether MYB could serve as a biomarker for cancer screening, prognosis prediction and accurate therapy design in various human cancers. In the present study, we validated that MYB is up-regulated in bladder cancer and overexpression of MYB significantly reinforced the cell migration ability of bladder cancer cells. However, it is unclarified whether MYB affects different tumor immune microenvironments and the pathogenic role of MYB in various cancer types still remains unclear. Our team aimed to exhibit the association between MYB and tumor progression and TIME for determining whether MYB could serve as a biomarker for cancer screening, prognosis and accurate therapy design.

## Materials and methods

### Cell lines, cell culture and transfection

SV-HUC-1, T24 and UM-UC3 cell lines were purchased from the Cell Bank of Type Culture Collection of the Chinese Academy of Sciences (Shanghai, China). RPMI 1640 medium was used to culture T24 cells. UM-UC3 and SV-HUC-1 cells were cultured in MEM. The heat-inactivated fetal bovine serum (10%) was added to the media mentioned above. All applied cell lines were cultured at a temperature of 37°C under an atmosphere of 5% CO_2_. All the analyzed samples were at the same passage. The MYB overexpression plasmids and control null plasmids (vector CMV-MCS-3FLAG-SV40-Neomycin) were transfected into cells by FuGene HD transfection reagent (Promega) according to the manufacturer’s instructions.

### RNA isolation and qRT-PCR

RNA was extracted from cell lines with RNAiso plus (TaKaRa) and reversely transcribed into cDNA with the PrimeScript RT reagent Kit. SYBR Premix Ex Taq (TaKaRa) was used to quantify the transcribed cDNA on the ABI 7500 fast real-time PCR System (Carlsbad, U.S.A.). GAPDH mRNA were used as endogenous references to calculate the relative expression of associated genes with the 2^−ΔΔCt^ method. Three duplicate samples were analyzed in each group. All primers used are listed as follows: GAPDH: 5′-CGGATTTGGTCGTATTGGG-3′ (forward) and 5′-CTGGAAGATGGTGATGGGATT-3′ (reverse); MYB: 5′-GACCCGGGAAGAGGATGAAA-3′ (forward) and 5′-CTCCCCTTTAAGTGCTTGGC-3′ (reverse).

### Wound healing assay

After transfection, cells were grown to 100% confluency in six-well plates and cross wounds were made using micropipette tips. Wound healing photograph was obtained after 24 h of culture by phase-contrast microscopy (Olympus). The number of cells were counted directly with photographs. The UM-UC3 cells lack adequate migration ability, thus was not suitable for wound healing assay.

### Transwell assay

Transwell assay was used to assess cell migration using transwell chambers (Millipore). After transfection, approximately 5 × 10^4^ T24 cells or 8 × 10^4^ UM-UC3 cells were resuspended in 200 µl of serum-free MEM and distributed on to the upper layer of the chambers. Then, we place the entire chamber into a 24-well plate and add 600 µl of medium supplemented with 10% FBS to the lower compartment. After culturation for 24 h at 37°C, chambers were treated with methanol and 0.1% crystal violet. Cells on the top surface of the membrane were carefully removed with a cotton swab. Photographs were acquired by a phase-contrast microscope (CARL ZEISS) using a 20× objective.

### Statistical analysis

The statistical analysis was conducted using Prism 7 (GraphPad Software, Inc., La Jolla, CA). All the experiment data was obtained from at least three independent experiments. Statistical significance was calculated by two-tailed unpaired Student’s *t*-test. *P*<0.05 was considered significant.

### Data source

Gene expression data, clinical phenotype data and mutation data for 33 cancers were downloaded from the UCSC Xena database (https://xena.ucsc.edu/). Data from TCGA and GTEx databases was also gathered for analysis. These databases included transcriptomic data for tumors and normal samples. The analysis was performed via SangerBox (http://sangerbox.com/tool.html) to process the raw data from UCSC Xena database. In order to convert RNA-seq data from FPKM format into TPM format, log2 conversion was performed.

### Topology, localization and protein interaction network of MYB proteins

Human Protein Atlas database (http://www.proteinatlas.org) is an open-access resource for analyzing protein expression in tissues, single cells, immune cells, cell lines and other subcellular levels. In the Human Protein Atlas database, the protein subcellular localization of MYB was obtained and analyzed in HEL, REH and U-2 OS cell lines. The association between MYB expression, endoplasmic reticulum, microtubules and the nucleus was investigated in the database. PROTTER (http://wlab.ethz.ch/protter/start/) is an open-access tool for predicting sequence features as well as protein-form visualization based on experimental proteomics evidence. String (cn.string-db.org/) is an open-source database which was utilized to predict protein–protein interaction (PPI) networks (confidence score cutoff = 0.7).

### Expression and prognostic value of MYB in pan-cancer

The TIMER database (https://cistrome.shinyapps.io/timer/) and UCSC Xena database were used to compare MYB expression between human cancers and paired normal tissue. Expression profiles of MYB were analyzed in different cancer lines and paired normal lines using the BioGPS database (http://biogps.org). We used UCSC Xena database and SangerBox tools to analyze the impact of the expression level of MYB on the overall survival of patients. The Kaplan–Meier method and univariate Cox regression analysis were used for survival analyses.

### Analyzing the immune and molecular subtypes related to MYB expression

TISIDB (http://cis.hku.hk/TISIDB/index.php) integrates multiple heterogeneous data types for tumor-immune system interaction. It was used to examine the correlation between MYB expression and immune or molecular subtypes of different cancer types. *P*<0.05 was considered to be statistically significant.

### Analyzing the correlation between MYB expression and immune checkpoint genes

The correlation between MYB expression and immune checkpoint genes was analyzed via UCSC Xena database. The immune checkpoint genes were classified into Inhibitory genes and stimulatory genes according to a previous publication [19].

### Analyzing tumor immune microenvironment and MYB in pan-cancer

SangerBox tools was utilized to calculate the stromal score, immune score and ESTIMATE score in human cancers. Only results with *P*<0.05 were considered for analysis. Further, the Timer method of IOBR package was used to re-evaluate the B cell, T cell CD4, T cell CD8, Neutrophil, Macrophage and DC invasion score of each patient in each tumor according to gene expression of MYB [20,21].

### Analyzing the relationship between MYB gene alteration and tumor immunity

Using the Tumor Immune Dysfunction and Exclusion Database (TIDE), immune checkpoint blockade treatment could be predicted (http://tide.dfci.harvard.edu/). The MYB predictive value was examined in TIDE compared with some previously published predictive markers in immune checkpoint blockade cohorts and the relationship between MYB expression and responses to immune checkpoint blockade treatments and T-cell dysfunction levels were evaluated in diverse cohorts. The cBioportal database analyzed MYB copy number alterations and genetic alterations within different cancers (http://www.cbioportal.org/).

## Results

### MYB is up-regulated in bladder cancer

**Figure 1:**
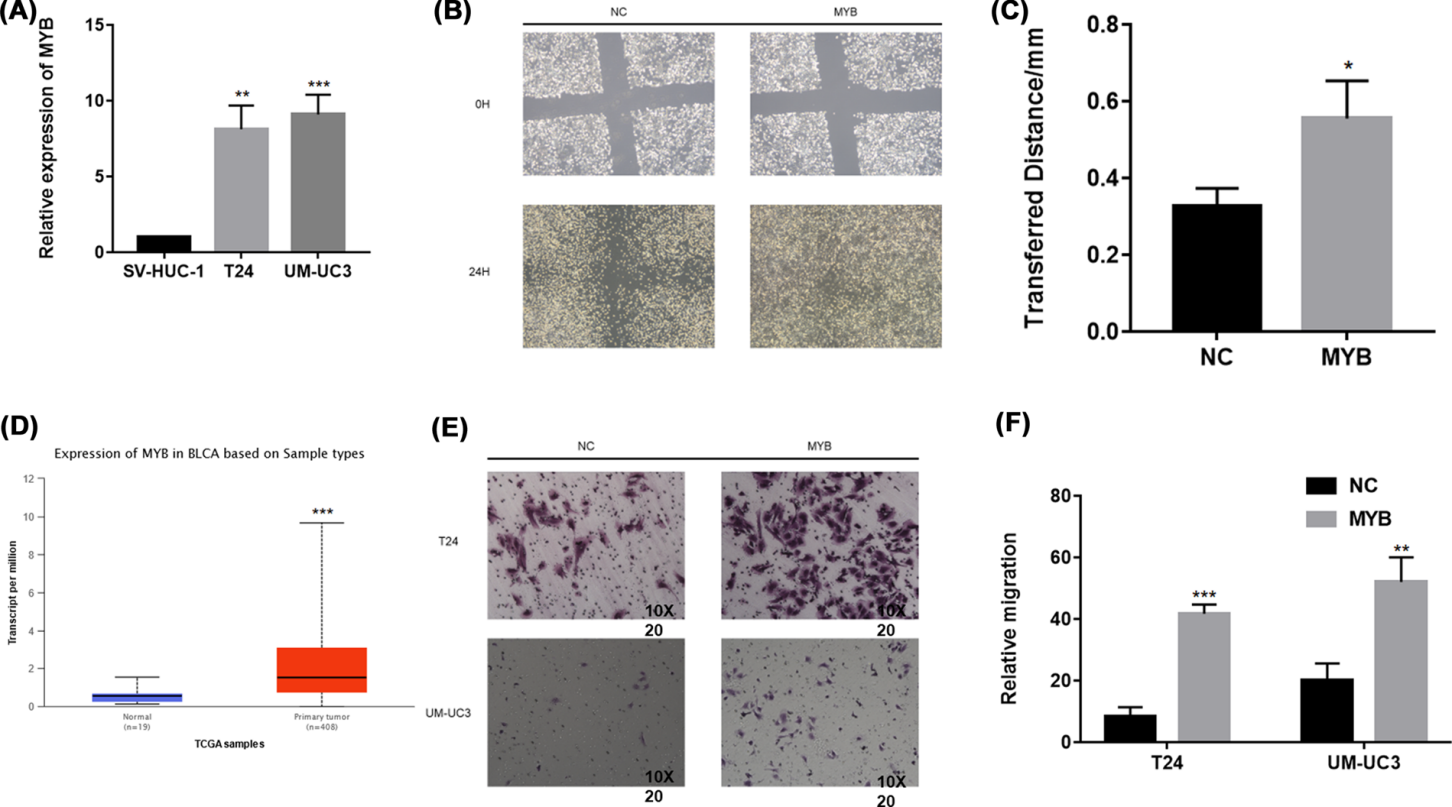
Biological function of MYB in bladder cancer (**A**) The relative expression of MYB is confirmed by qRT-PCR in SV-HUC-1, T24 and UM-UC3 cells. (**B**) The wound healing assay demonstrated a significant enhancement of T24migration ability. (**C**) The transferred distance of T24 was calculated in three independent experiments. (**D**) The expression of MYB was explored in UALCAN database. (**E**) The representative micrographs of transwell assays in T24 and UM-UC3 after MYB overexpression. The photographs were taken under 10*20 scope. (**F**) The representative micrographs of transwell assays were calculated. Error bars represent the S.D. obtained from three independent experiments; *P<0.05, **P<0.01, ***P<0.001.

Quantitative real-time PCR (qRT-PCR) was conducted to verify the expression level of MYB in bladder cancer cell lines. The results indicated a higher expression level of MYB in two bladder cancer cell lines, T24 and UM-UC3, compared with that in the normal urothelial cell line, SV-HUC-1 (Figure 1A). Then, we explored the expression of MYB in UALCAN database (http://ualcan.path.uab.edu/) to determine that the expression of MYB is higher in bladder cancer (Figure 1D).

### Overexpression of MYB enhanced the migration of bladder cancer

Next, we suspected whether MYB played an oncogenic role in the progression of bladder cancer. After transfection, we first performed the CCK-8 and colony formation assays to detect the effects of MYB on cell proliferation, however, no positive results were found (data shown in supplementary materials). Interestingly, we validated a significant enhancement of cell mobility resulting from MYB overexpression by wound healing assay (Figure 1B,C) and transwell assay (Figure 1E,F).

### Topology, localization and protein interaction network of MYB proteins

**Figure 2:**
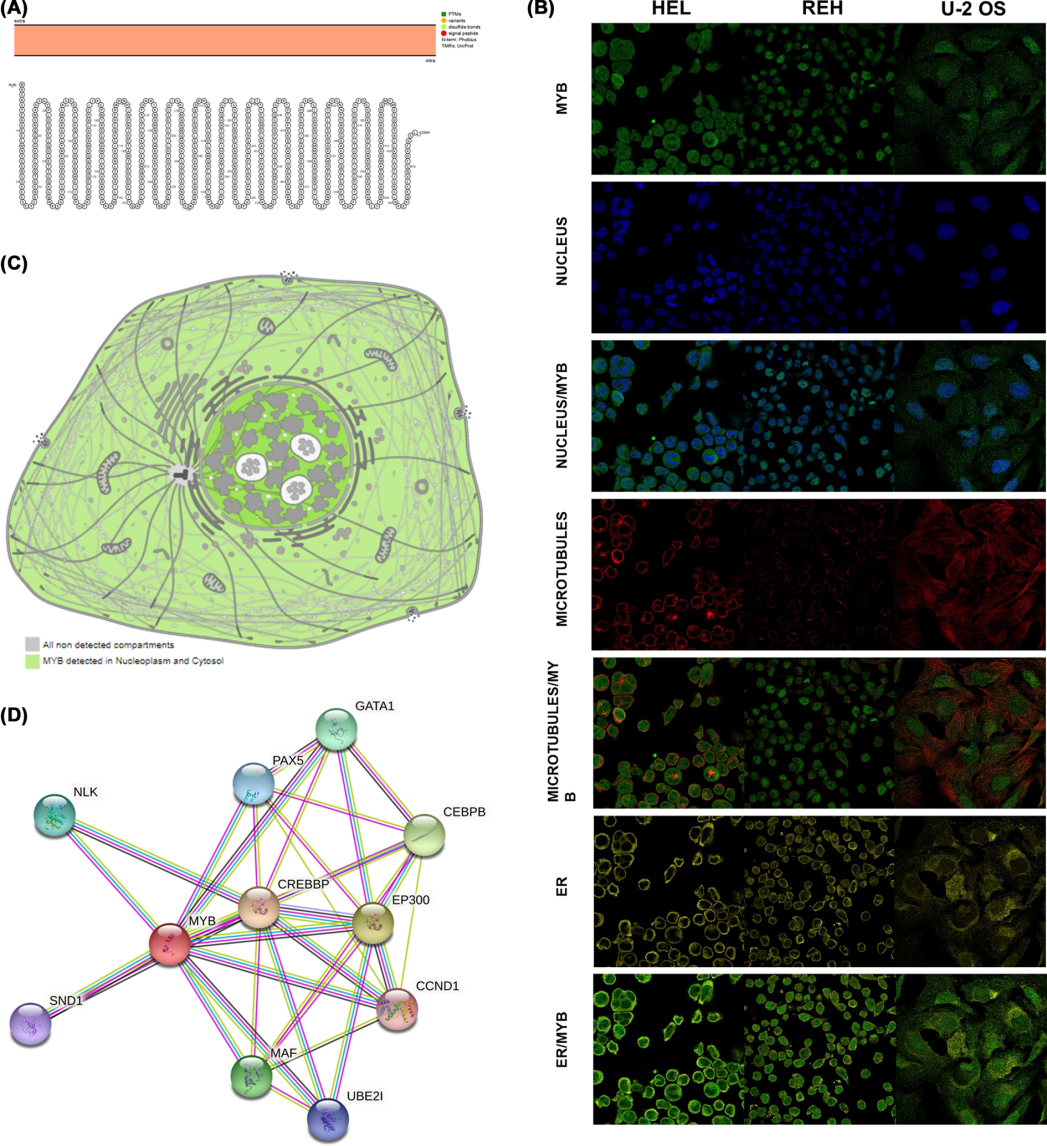
Topology, localization and protein interaction network of MYB proteins (**A**) Topology of MYB proteins indicating intracellular membrane localization. (**B**) Immunofluorescence staining of the intracellular protein localization of MYB in the nucleus, microtubules AND endoplasmic reticulum of HEL, REH and U-2 OS cell lines was explored from the Human Protein Atlas database. (**C**) The expression of MYB was mainly distributed at the nucleoplasm and cytosol. (**D**) Network of MYB protein interactions from String database.

To further explore the role of MYB in human cancer, we conducted a pan-cancer analysis of MYB. The fundamental role of proteins is always associated with its topology, localization and protein interaction network. Physiologically the topology of MYB displayed intracellular membrane localization (Figure 2A). To further explore the intracellular protein localization of MYB, we retrieved data from The Human Protein Atlas database. As shown in Figure 2B, the location of protein MYB was heterogeneously distributed at nucleus, microtubes and ER in HEL, REH and U-2 OS cell lines. The sketch of cell intuitionally indicated that the MYB protein is mainly detected in nucleoplasm and cytosol (Figure 2C). Protein–protein interaction analysis revealed that the proteins associated with MYB mainly included NLK, SND1, GATA1, PAX5, CEBPB, CREBBP, EP300, CCND1, MAF and UBE2I (Figure 2D).

### Expression of MYB in pan-cancer

**Figure 3:**
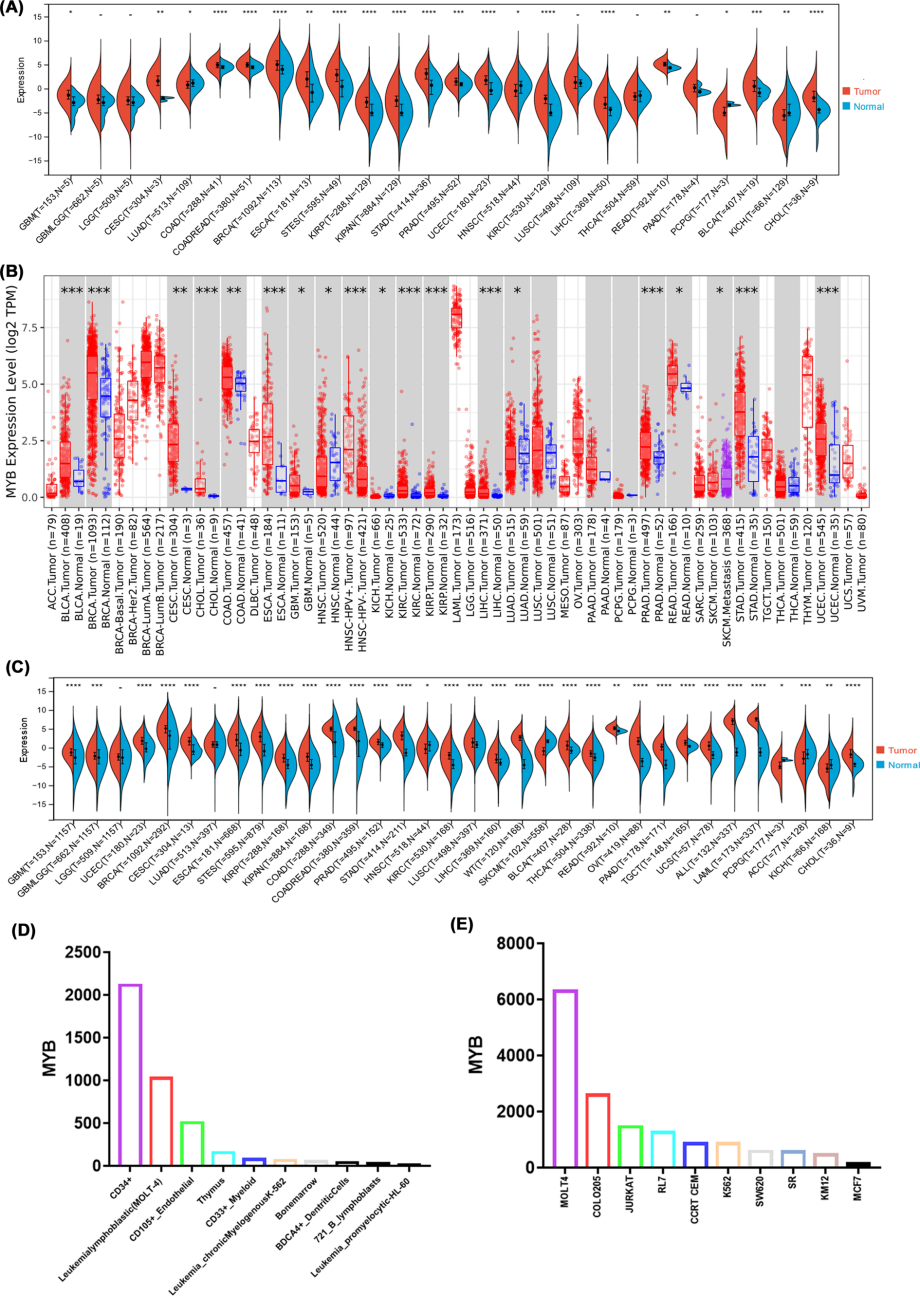
Expression of MYB in pan-cancer (**A**) The expression level of MYB in human cancers from TCGA. (**B**) The expression level of MYB was verified via TIMER 2.0 database which is based on TCGA database in human cancers. (**C**) The expression level of MYB in human cancers from TCGA and GTEx databases. (**D**) The expression level of MYB in normal tissues and cancer cell lines identified via BioGPS database; *P<0.05, **P<0.01, ***P<0.001.

To further study the relationship between MYB and cancers, we identified the mRNA expression level in different tissues using several databases. We used TCGA database to obtain the expression patterns of MYB in cancers and paraneoplastic tissues (Figure 3A). It can be found that the expression level of MYB is significantly higher in cancers including GBM, CESC, COAD, COADREAD, BRCA, ESCA, STES, KIRP, KIPAN, STAD, PRAD, UCEC, KIRC, LIHC, READ, BLCA and CHOL (*P*<0.05, cancer type abbreviations was shown in Supplementary Table S1). On the contrary, the expression of MYB is lower in cancers such as LUAD, HNSC, PCPG and KICH. Meanwhile we used TIMER 2.0 database, which is based on TCGA database to verify the expression of MYB in different cancers (Figure 3B). Next, we combined the data from TCGA and GTEx database (Figure 3C). It was found that expression of MYB is up-regulated in 27 cancers including GBM, GBMLGG, UCEC, BRCA, CESC, ESCA, STES, KIRP, KIPAN, COAD, COADREAD, PRAD, STAD, KIRC, LUSC, LIHC, WT, BLCA, THCA, READ, OV, PAAD, TGC, UCS, ALL, LAML and CHOL. While the expression of MYB is significantly lower in only 5 cancers including HNSC, SKCM, PCPG, ACC and KICH. Overall, MYB is overexpressed in most cancer types via the analysis of the data from TCGA and GTEx.

Additionally, the expression level of MYB in normal tissues and cancer cell lines was identified via BioGPS database. We displayed 10 normal tissues with the highest expression level of MYB in Figure 3D. Similarly, 10 cancer cell lines with the highest expression level of MYB was depicted in Figure 3E. The results above suggested that MYB expression level is higher in several immune cells and the expression of MYB in cancer cell lines is generally higher than normal tissues.

### Prognostic value of MYB in pan-cancer

**Figure 4:**
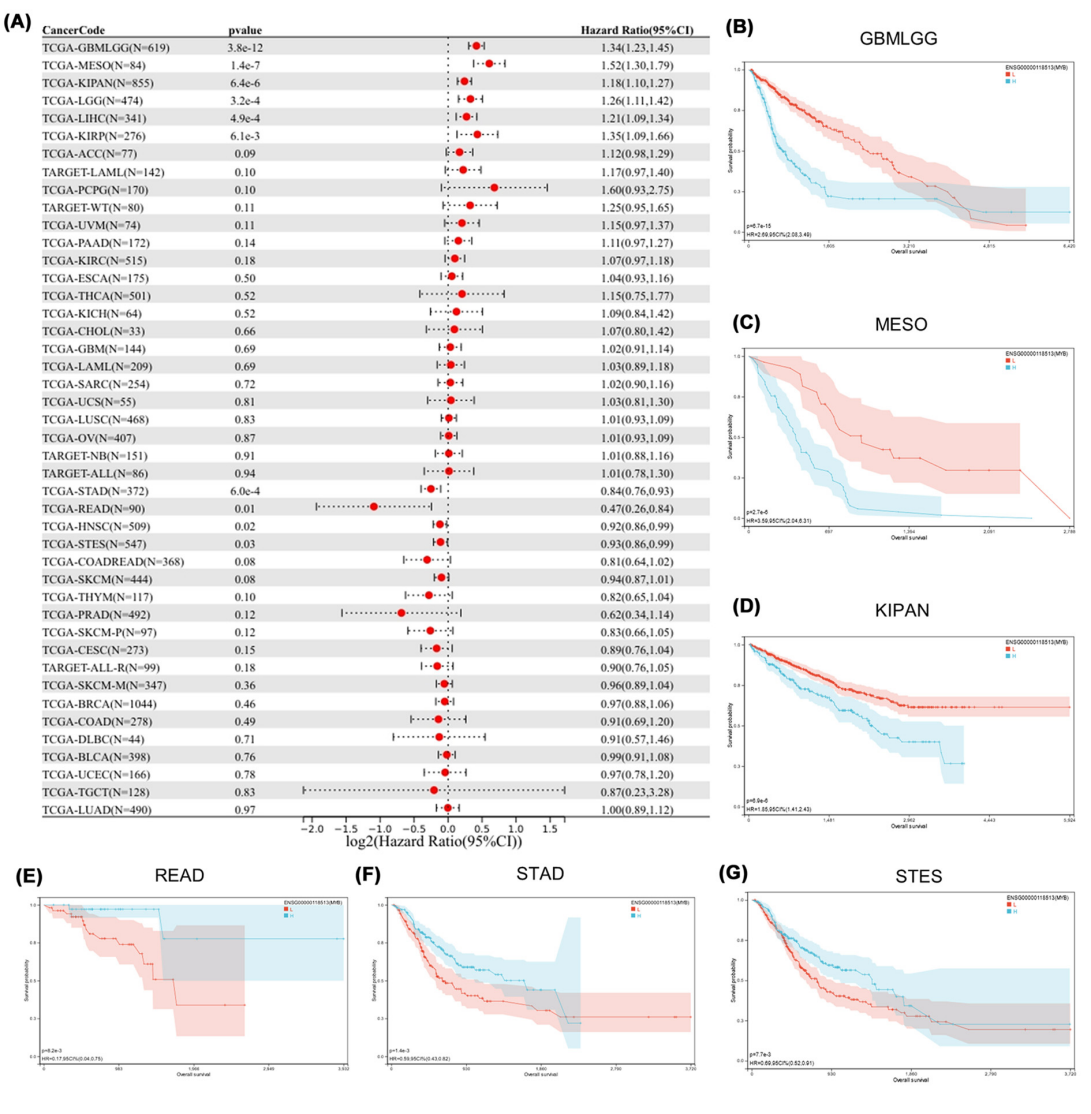
Prognostic value of MYB in pan-cancer (**A**) MYB expression and tumor OS in pan-cancer. (**B–G**) Kaplan–Meier analysis of MYB expression and OS in GBMLGG, LGG, KIRP, KIPAN, LIHC and TCGA-MESO.

We further evaluated the impact of the expression level of MYB on the clinical prognosis of patients with multiple cancers from UCSC Xena database. Cox regression model was utilized to analyze the Overall survival of each cancer (Figure 4A). It can be observed that higher expression of MYB was significantly associated with a poorer outcome in six cancers including GBMLGG, LGG, KIRP, KIPAN, LIHC and TCGA-MESO. On the contrary, lower expression of MYB was significantly associated with a poorer outcome in four cancers including STES, STAD, HNSC and READ. In addition, we conducted Kaplan–Meier analysis to verify the impact of MYB expression on patient prognosis in several cancers (Figure 4B–G). Together, MYB may act as a cancer-promoting gene or a cancer-suppressing gene in different cancer types.

### The immune and molecular subtypes related to MYB expression in pan-cancer

**Figure 5:**
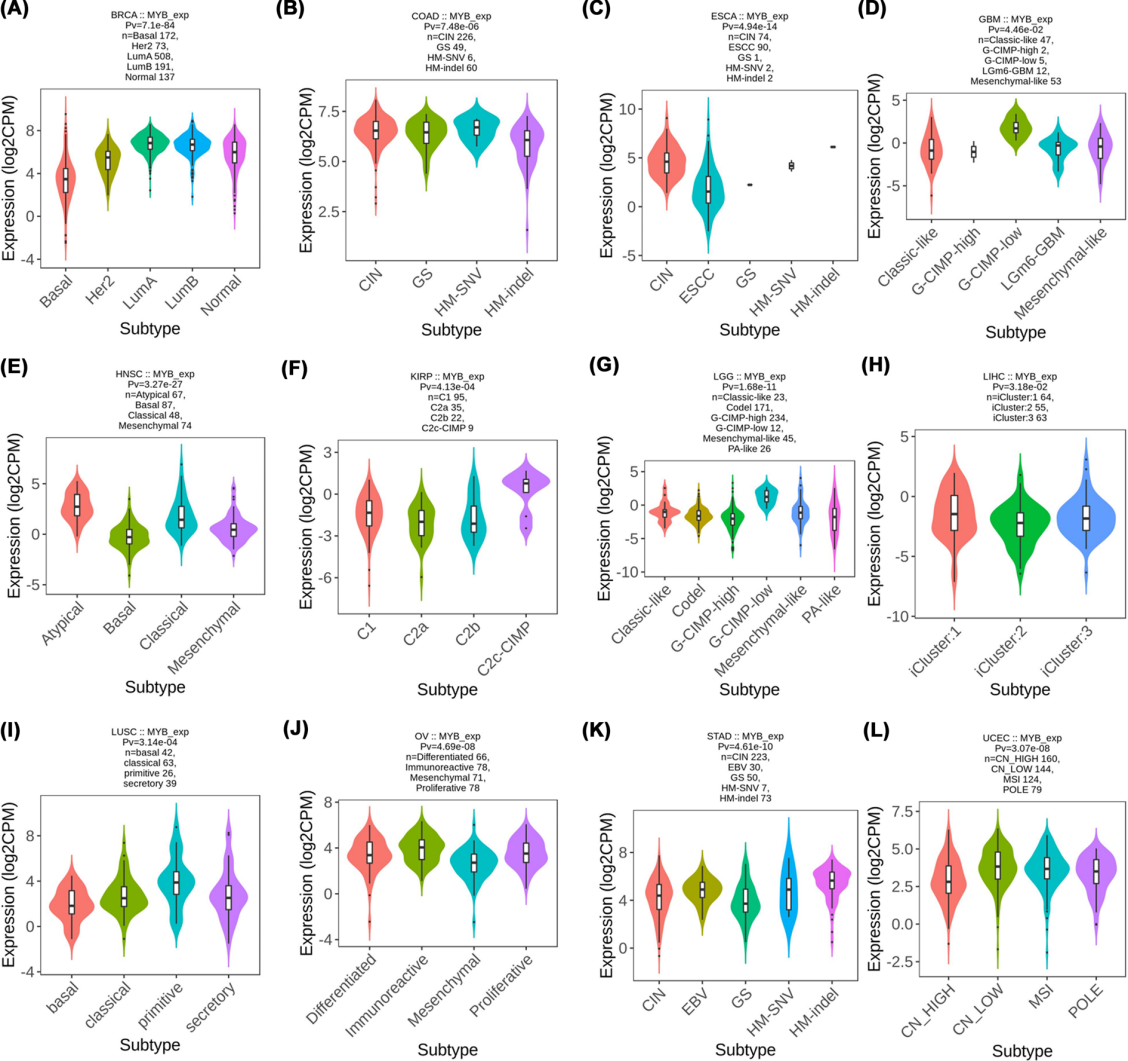
The association of MYB and molecular subtypes across human cancers (A) We analyzed the relationship of MYB and molecular subtypes in BRCA; (B) We analyzed the relationship of MYB and molecular subtypes in COAD; (C) We analyzed the relationship of MYB and molecular subtypes in ESCA; (D) We analyzed the relationship of MYB and molecular subtypes in GBM; (E) We analyzed the relationship of MYB and molecular subtypes in HNSC; (F) We analyzed the relationship of MYB and molecular subtypes in KIRP; (G) We analyzed the relationship of MYB and molecular subtypes in LGG; (H) We analyzed the relationship of MYB and molecular subtypes in LIHC; (I) We analyzed the relationship of MYB and molecular subtypes in LUSC; (J) We analyzed the relationship of MYB and molecular subtypes in OV; (K) We analyzed the relationship of MYB and molecular subtypes in STAD; (L) We analyzed the relationship of MYB and molecular subtypes in UCEC.

**Figure 6:**
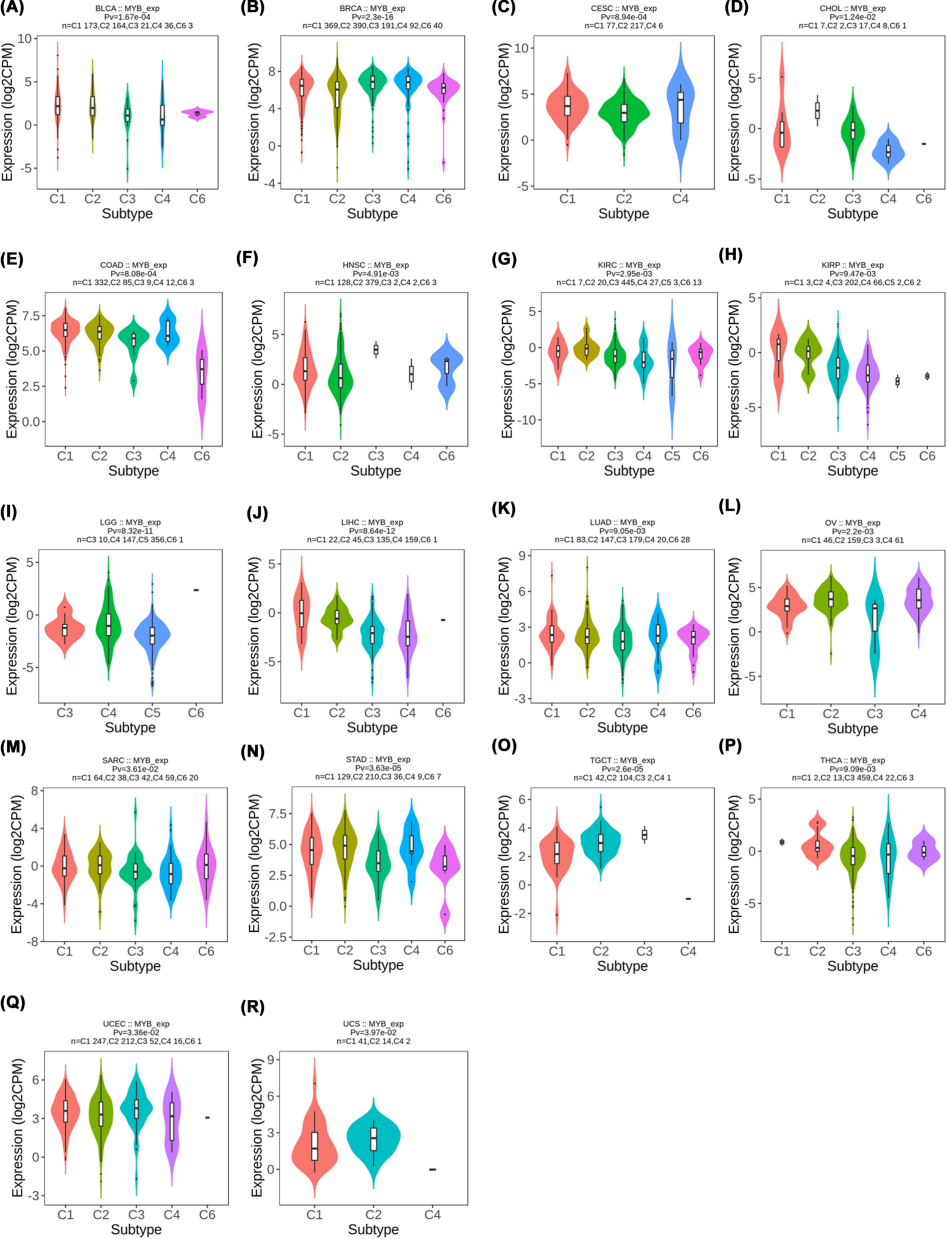
The association of MYB and immune subtypes across human cancers (A) We analyzed the relationship of MYB and immune subtypes in BLCA; (A) We analyzed the relationship of MYB and immune subtypes in BLCA; (B) We analyzed the relationship of MYB and immune subtypes in BRCA; (C) We analyzed the relationship of MYB and immune subtypes in CESC; (D) We analyzed the relationship of MYB and immune subtypes in COAD; (E) We analyzed the relationship of MYB and immune subtypes in COAD; (F) We analyzed the relationship of MYB and immune subtypes in HNSC; (G) We analyzed the relationship of MYB and immune subtypes in KIRC; (H) We analyzed the relationship of MYB and immune subtypes in KIRP; (I) We analyzed the relationship of MYB and immune subtypes in LGG; (J) We analyzed the relationship of MYB and immune subtypes in LIHC; (K) We analyzed the relationship of MYB and immune subtypes in LUAD; (L) We analyzed the relationship of MYB and immune subtypes in OV; (M) We analyzed the relationship of MYB and immune subtypes in SARC; (N) We analyzed the relationship of MYB and immune subtypes in STAD; (O) We analyzed the relationship of MYB and immune subtypes in TGCT; (P) We analyzed the relationship of MYB and immune subtypes in THCA; (Q) We analyzed the relationship of MYB and immune subtypes in UCEC; (R) We analyzed the relationship of MYB and immune subtypes in UCS.

We then utilized the TISIDB database to clarify the relationship between immune and molecular subtypes and MYB expression in different cancers [22]. A significant association between molecular subtype and MYB expression was detected in BRCA, COAD, ESCA, GBM, HNSC, KIRP, LGG, LIHC, LUSC, OV, STAD and UCEC (Figure 5). For the six immune subtypes (C1-wound healing, C2-IFN-γ dominant, C3-inflammatory, C4 -lymphocyte depleted, C5-immunologically quiet and C6-TGF-b dominant) of cancers, a significant connection with MYB expression was found in 18 types of cancer including BLCA, BRCA, CESC, CHOL, COAD, HNSC, KIRC, KIRP, LGG, LIHC, LUAD, OV, SARC, STAD, TGCT, THCA, UCEC and UCS (Figure 6). Interestingly, we found that MYB expression has a significant relationship with both immune and molecular subtypes in several cancer types such as BRCA, COAD, HNSC, KIRP, LGG, LIHC, OV, STAD and UCEC, strongly suggesting that MYB may play a key role in these cancers and further studies are needed to verify the potential of MYB for becoming an ideal target of precise treatment.

### Immune check point genes related to MYB expression in pan-cancer

**Figure 7:**
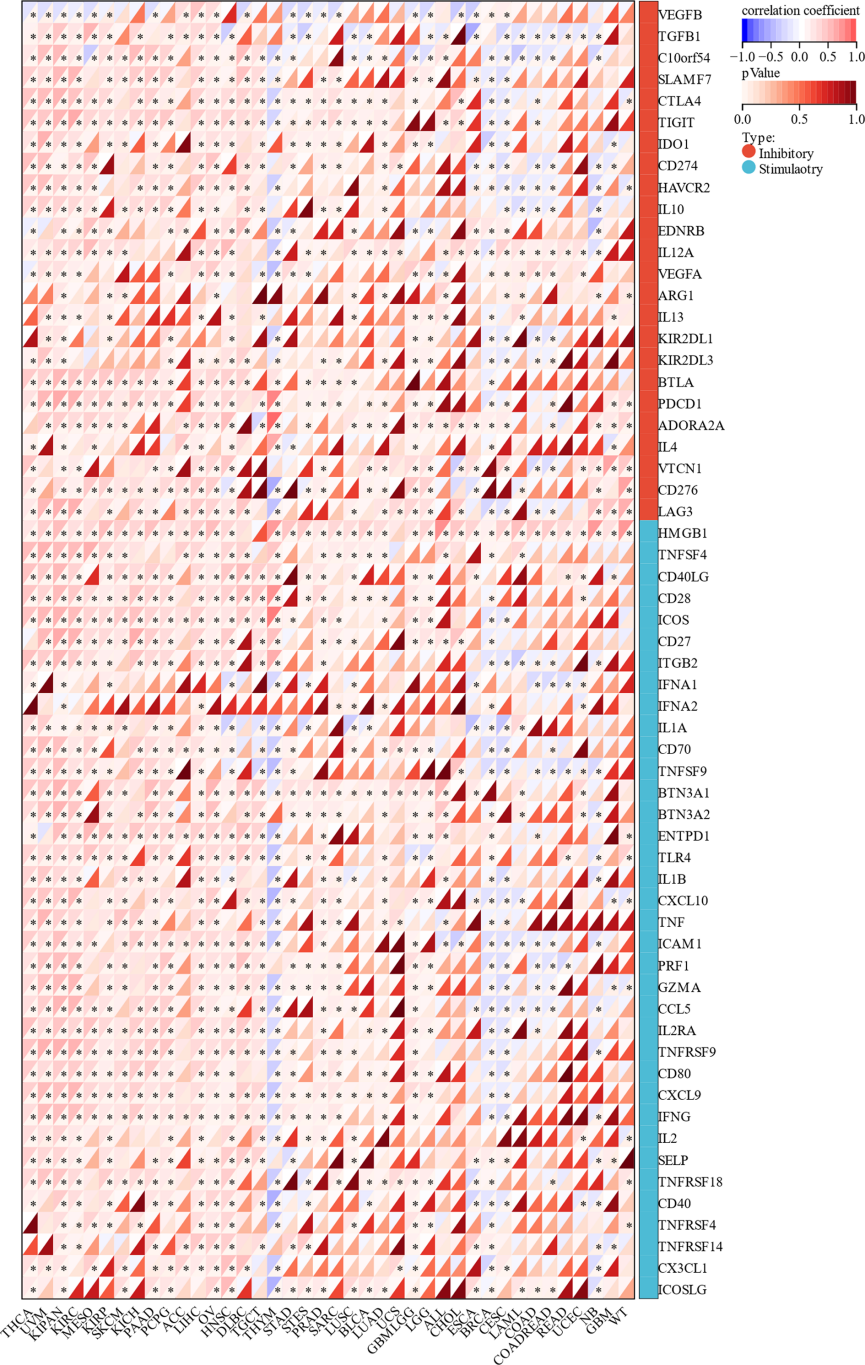
Correlation between MYB and immune checkpoint genes, including 24 inhibitory genes and 36 stimulatory genes in 40 human cancers *P < 0.05; **P < 0.01; ***P < 0.001.

The status of immune check point genes plays a key role in cancer immune cell infiltration and immunotherapy. Therefore, we studied the relationship between MYB expression and immune checkpoint genes in human cancers to access the potential of MYB in immunotherapy. We downloaded data from UCSC Xena database and explored the expression of MYB and two types of immune checkpoint genes, including 24 inhibitory genes and 36 stimulatory genes in 40 human cancers. The correlation of MYB and immune checkpoint genes was calculated and displayed in Figure 7. A significant relationship was found between MYB and most of immune checkpoint genes in a number of cancers such as THCA, UVM, KIPAN, KIRC, MESO, KIRP, SKCM, KICH, PAAD, PCPG, ACC, LIHC, OV, HNSC, DLBC, TGCT, THYM, STAD, STES and PRAD. Especially in THCA, 52 in a total of 60 immune checkpoint genes has a significant correlation with MYB expression. This result suggests that MYB may regulate the expression of these immune checkpoint genes in different pathways, and thus exert potential effect on immunotherapy. Overall, our analysis indicated that MYB may act as a potential biomarker for predicting the outcome of immunotherapy or as a new target to improve the effect of immunotherapy.

### Tumor immune microenvironment and MYB in pan-cancer

**Figure 8:**
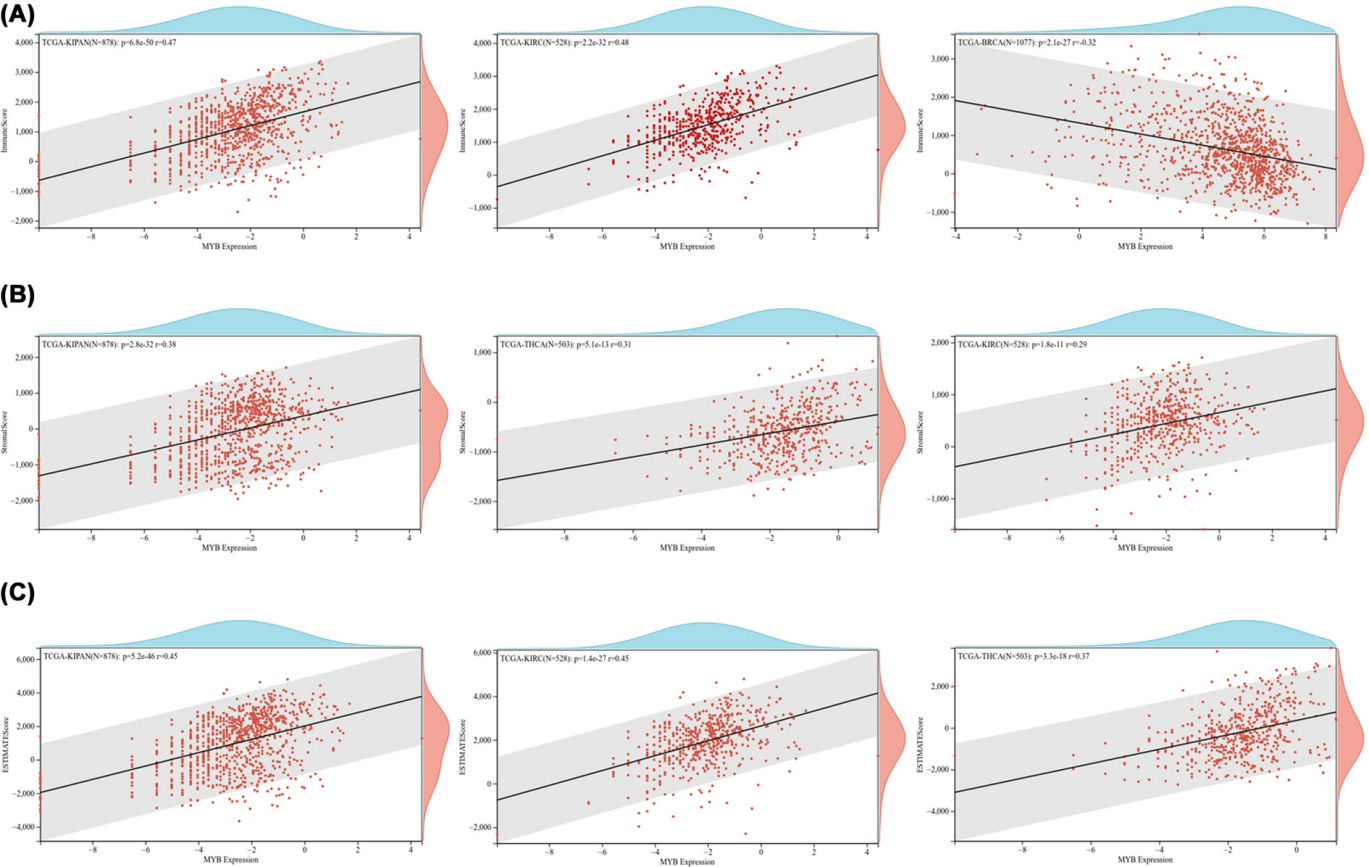
The associations between MYB expression and immune infiltration scores in human cancers (A) The top three tumors with the most significant association between MYB expression and immune score was displayed; (B) The top three tumors with the most significant association between MYB expression and stromal score was displayed; (C) The top three tumors with the most significant association between MYB expression and ESTIMATE score was displayed.

**Figure 9:**
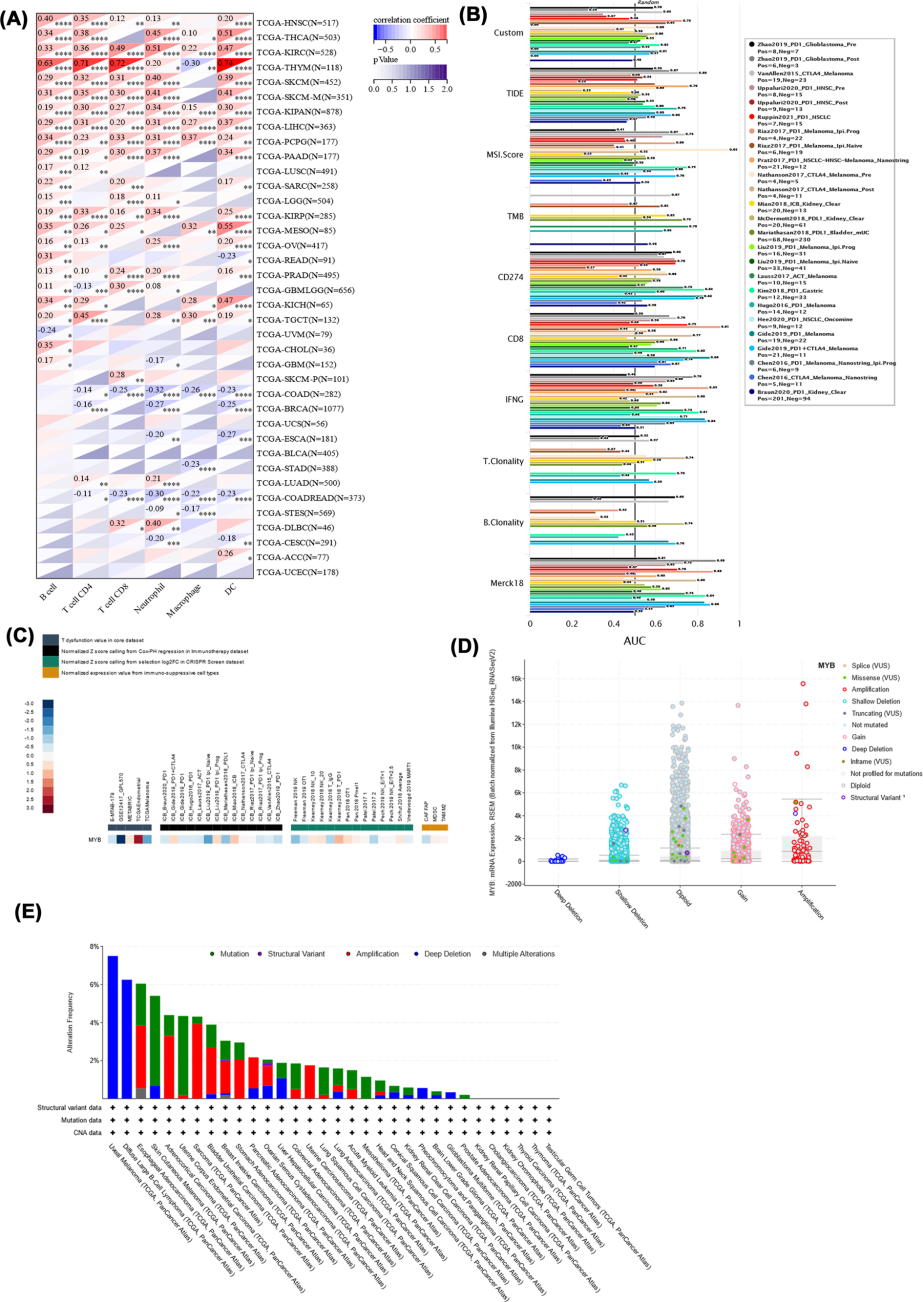
MYB gene alteration and relationship with tumor immunity (A) The potential relationship between MYB expression and infiltration of immune cells. (B) The value of MYB as a biomarker comparing with traditional immunotherapy biomarkers. (C) The association between MYB expression with T cell dysfunction, immunotherapy outcome, natural killer cell anti-tumor activity in CRISPR screen dataset and expression value from immune-suppressive cell types. (D) The copy-number alterations of MYB. Amplification was the most common type of copy-number alteration in MYB. (E) The genomic alteration of MYB. Deep deletion was the most frequent alteration type.

Emerging evidence illustrated that tumor immune microenvironment played a significant role in the occurrence and progression of human cancers. Hence, we explored the relationship between MYB expression and immune signatures by calculating stromal score, immune score and ESTIMATE score. Among the 44 types of cancer, we found that the top three tumors with the most significant association between MYB expression and immune score were KIPAN, KIRC and BRCA (Figure 8A). Similarly, the top three tumors with the most significant association between MYB expression and stromal score were identified as KIPAN, THCA and KIRC (Figure 8B). Surprisingly, the top three tumors with the most significant association between MYB expression and ESTIMATE score were the same as stromal score including KIPAN, THCA and KIRC (Figure 8C). These findings strongly suggest that MYB expression levels in KIPAN, THCA and KIRC were positively connected with the infiltration of immune cells and stromal cells. Furthermore, we analyzed the potential relationship between MYB expression and infiltration of several important types of immune cells (Figure 9A). In a total of 38 types of human cancer, MYB expression had a significant relationship with cell infiltration in 31 types of human cancer. MYB had a strong correlation with B cell in 24 cancer types, CD4 T cell in 22 cancer types, CD8 T cell in 18 cancer types, neutrophil in 25 cancer types, macrophage in 13 cancer types and DC in 24 cancer types.

### MYB gene alteration and relationship with tumor immunity

We examined the value of MYB as a biomarker through comparing it with traditional immunotherapy biomarkers according to their different outcome after immune check point blockade therapy via TIDE dataset. The results illustrated that MYB as an immunotherapy biomarker had an area under the receiver operating characteristic curve (AUC) which is greater than 0.5 in 12 of the 25 immunotherapy cohorts (Figure 9B). It is inspiring to find that MYB had a similar predictive ability with MSI.Score, and the predictive value of MYB may be superior to TMB, T. Clonality and B. Clonality.

Next, we assessed the association between MYB expression and immunotherapy outcome in several cohorts (Figure 9C). High MYB expression is related to worse outcomes of anti-PD-1+ anti-CTLA4 treatment in melanoma, while received good outcomes of anti-PD-1 treatment in kidney cancer, melanoma, bladder cancer and glioblastoma. Meanwhile, results of T-cell dysfunction value in core dataset revealed a strong positive relationship between MYB expression and T-cell dysfunction value in endometrial cancer and a strong negative relationship in leukemia. Interestingly, we found that MYB-knockout in CRISPR screen dataset limited natural killer cell antitumor activity in melanoma but enhanced natural killer cell activity in colon adenocarcinoma. At the same time, low-expression level of MYB was detected in FAP (+) tumor-associated fibroblasts by microarray.

We also evaluated the genomic alteration of MYB through cBioPortal dataset. Deep deletion was the most frequent alteration type of MYB, followed by mutation and amplification (Figure 9E). Subsequently, the copy-number alterations of MYB were analyzed (Figure 9D). Amplification was the most common type of copy-number alteration in MYB, followed by gene gain, diploid, shallow deletion and deep deletion.

## Discussion

It is becoming increasingly clear that MYB is significantly linked to the growth and metastasis in a wide range of malignancies. As a transcription factor, MYB broadly participate in the transcription process to transactivate gene expression via binding the same DNA consensus sequence [PyAAC(G/T)G] [18]. We first explored the effect of MYB in bladder cancer via basic experiments and validated that MYB is up-regulated in bladder cancer and overexpression of MYB significantly reinforced the cell migration ability of bladder cancer cells. This result convinced us that MYB may act as an oncogene in human cancer and we further conducted a pan-cancer analysis to verify the function of MYB in human cancers. Of the 34 tumors we examined in GTEx and TCGA databases, MYB gene expression level was significantly higher than normal tissues in 27 kinds of tumors and lower in 5 kinds of tumors. Meanwhile, several previous studies determined our result on the relationship between MYB and tumors. In breast cancer, MYB acts as a promoter of breast cancer metastasis via activating the Wnt/β-catenin/Axin2 signaling pathway [23]. Qu et al. reported that in colorectal cancer, MYB promotes tumor progress by enhancing c-fos-induced epithelial–mesenchymal transition [24]. Another study revealed that in renal cancer MYB activated the transcription of miR-520h, which targeted MAGI1 and down-regulated its expression to suppress invasiveness and metastasis of tumor [25]. In contrast, some researchers reported different results. For example, Zhao et al. found that MYB is highly expressed in laryngeal squamous cell carcinoma (consists approximately 90% of head and neck squamous cell carcinoma) and induced miR-155 expression to promote cancer proliferation, invasiveness and migration [26]. However, our data from GTEx and TCGA databases indicated that MYB expression is significantly lower in head and neck squamous cell carcinoma and lower expression of MYB predicts poorer prognosis, suggesting that MYB acted as a tumor suppresser in head and neck squamous cell carcinoma. This contradiction may largely be caused by tumor heterogeneity, implying that the spatiotemporal specify of MYB RNA may decide and lead to diverse cell fate in tumor genesis and progression. These findings suggested that MYB may be a potential biomarker to predict the prognosis of different malignancies while more studies are necessary to further determine the exact role of MYB in human cancers.

To identify the potential role of MYB in tumor progression and immunity, we explored the expression of MYB in different molecular subtypes and immune subtypes in human cancers. The results proved that MYB expression showed significant difference among several immune subtypes and molecular subtypes in most cancers, which could demonstrate that MYB is a valuable pan-cancer diagnostic biomarker and involved in the tumor immune environment regulation. In order to further identify the association between MYB and tumor immune environment, we calculated the immune infiltration score and explored the immune cell infiltration. According to our analysis, in a series of cancer types the expression level of MYB is significantly related to immune score and in approximately a half of these cancer types the MYB expression is positively related to immune score. This result suggests that MYB is correlated to tumor immune microenvironment in human cancers. Next, we analyzed the exact immune cell infiltration in tumor immune microenvironment and found that MYB expression had a strong correlation with B cell, CD4 T cell, neutrophil and DC in most cancer types. Tumor-infiltrating B cell mediates antitumor immunity via promoting maturation of tumor-associated tertiary lymphoid structures, while B cells and mature tertiary lymphoid structures is an important predictor of immunotherapy response [27]. CD4 T cell, along with specific DCs, help enhance the magnitude and quality of the activity of cytotoxic T lymphocytes, which established high-efficiency antitumor immunity [28]. Considering that the relationship between immune cells and MYB expression is diverse in human cancers, we infer there is an intricate correlation between the antitumor or pro-tumor response of immune cells and MYB expression. Meanwhile, the correlation of MYB and immune checkpoint genes indicated that MYB may regulate tumor immunity in complex molecular pathways. Furthermore, MYB as an immunotherapy biomarker is superior to traditional immunotherapy biomarkers involving TMB, T. Clonality and B. Clonality in half of included immunotherapy cohorts. Moreover, lower expression level of MYB was detected in FAP (+) tumor-associated fibroblasts, suggesting a potential relationship between tumor microenvironment and MYB. Overall, we found that MYB is related to tumor immunity in human cancers and may act as a novel predictor of immunotherapy. Further studies are needed to identify the molecular mechanisms of MYB in tumor progression and immunity.

Gene alteration analyze of MYB was also conducted in our study. As a result, deep deletion was the most frequent alteration type of MYB, followed by mutation and amplification. Subsequently, the copy-number alterations of MYB were analyzed. Amplification was the most common type of copy-number alterations in MYB, followed by gene gain, diploid, shallow deletion and deep deletion. Copy-number alterations are a vital component of genetic diversity and may contribute to rapid adaptive development and progression of human heritable and somatic diseases such as cancer [29].

Although we have comprehensively exhibited the prognostic and immunological roles of MYB in pan-cancer, we have to admit that some limitations still remain in our study. First, we only analyzed open-source data and we are unable to include all the data published, so the publication bias and selection bias are unavoidable. Second, there is heterogeneity between diverse databases and some contradictory results needed further affirmation. Third, we failed to confirm the role of MYB in most human tumors through experiments.

## Conclusion

We confirmed that the expression level of MYB is significantly higher in bladder cancer cell lines than in urothelial cells through qRT-PCR. Further experiments validated that overexpression of MYB enhanced the ability of migration in bladder cancer via wound healing assay and transwell assay. Based on our pan-cancer analysis of MYB, we found that MYB was differentially expressed between tumors and normal tissues, and there was a connection between MYB expression and prognosis. We conclude that MYB may serve as a powerful biomarker for tumor screening, prognostic, individualized treatment strategy in a broad range of malignancies. Moreover, MYB expression is related to the tumor microenvironment and immune cell infiltration in different cancer types. Different types of tumors respond differently to its effects on tumor immunity. In the future, more precise and personalized immunotherapy may be achieved by elucidating the role of MYB in tumor development.

## Supplementary Material

Supplementary Figure S1

Supplementary Table S1

## Data Availability

Some database that support the findings of this study are openly available described in ‘Materials and methods’, including UCSC Xena database [30], UALCAN [31], TCGA [32], GTEx [33], human protein atlas (www.proteinatlas.org), PROTTER [34], String [35], TIMER [36], BioGPS [37], TISIDB [22], TIDE [38] and cBioportal database [39,40]. Other data are available from corresponding author.

## Abbreviations

AML: acute myeloid leukemia
PPI: protein–protein interaction
TIDE: Tumor Immune Dysfunction and Exclusion Database
TIME: Tumor cells and tumor immune microenvironment

## References

[1] Siegel R.L., Miller K.D., Fuchs H.E. and Jemal A. (2022) Cancer statistics, 2022. CA Cancer J. Clin. 72, 1, 7–33 10.3322/caac.21708 35020204

[2] Yan H., Zhang L., Cui X., Zheng S. and Li R. (2022) Roles and mechanisms of the m(6)A reader YTHDC1 in biological processes and diseases. 8, 237 10.1038/s41420-022-01040-2 35501308 PMC9061745

[3] Yan H., Cui X., Zhang P. and Li R. (2021) Fruit and vegetable consumption and the risk of prostate cancer: a systematic review and meta-analysis. Nutr. Cancer 74, 1235–1242 10.1080/01635581. 34286657

[4] Cercek A., Lumish M., Sinopoli J., Weiss J., Shia J., Lamendola-Essel M. et al. (2022) PD-1 blockade in mismatch repair–deficient, locally advanced rectal cancer. N. Engl. J. Med. 386, 2363–2376 10.1056/NEJMoa2201445 35660797 PMC9492301

[5] Kalbasi A. and Ribas A. (2020) Tumour-intrinsic resistance to immune checkpoint blockade. 20, 25–39 10.1038/s41577-019-0218-4 31570880 PMC8499690

[6] Petitprez F., Meylan M., de Reyniès A., Sautès-Fridman C. and Fridman W.H. (2020) The tumor microenvironment in the response to immune checkpoint blockade therapies. Front. Immunol. 11, 784 10.3389/fimmu.2020.00784 32457745 PMC7221158

[7] Lv B., Wang Y., Ma D., Cheng W., Liu J., Yong T. et al. (2022) Immunotherapy: reshape the tumor immune microenvironment. Front. Immunol. 13, 844142 10.3389/fimmu.2022.844142 35874717 PMC9299092

[8] Tsui C., Kretschmer L., Rapelius S., Gabriel S.S., Chisanga D., Knöpper K. et al. (2022) MYB orchestrates T cell exhaustion and response to checkpoint inhibition. Nature 609, 354–360 10.1038/s41586-022-05105-1 35978192 PMC9452299

[9] Cicirò Y. and Sala A. (2021) MYB oncoproteins: emerging players and potential therapeutic targets in human cancer. Oncogenesis 10, 19 10.1038/s41389-021-00309-y 33637673 PMC7910556

[10] Walf-Vorderwülbecke V., Pearce K., Brooks T., Hubank M., van den Heuvel-Eibrink M.M., Zwaan C.M. et al. (2018) Targeting acute myeloid leukemia by drug-induced c-MYB degradation. Leukemia 32, 882–889 10.1038/leu.2017.317 29089643

[11] Yang R.M., Nanayakkara D., Kalimutho M., Mitra P., Khanna K.K., Dray E. et al. (2019) MYB regulates the DNA damage response and components of the homology-directed repair pathway in human estrogen receptor-positive breast cancer cells. Oncogene 38, 5239–5249 10.1038/s41388-019-0789-3 30971760

[12] Zhang L., Wang X., Li Y., Han J., Gao X. and Li S. (2021) c-Myb facilitates immune escape of esophageal adenocarcinoma cells through the miR-145-5p/SPOP/PD-L1 axis. Clinical and Translational Medicine 11, e464 10.1002/ctm2.464 34586738 PMC8473478

[13] Miree O., Srivastava S.K., Khan M.A., Sameeta F., Acharya S., Ndetan H. et al. (2021) Clinicopathologic significance and race-specific prognostic association of MYB overexpression in ovarian cancer. Sci. Rep. 11, 12901 10.1038/s41598-021-92352-3 34145334 PMC8213794

[14] Xu L.H., Zhao F., Yang W.W., Chen C.W., Du Z.H., Fu M. et al. (2019) MYB promotes the growth and metastasis of salivary adenoid cystic carcinoma. Int. J. Oncol. 54, 1579–1590 10.3892/ijo.2019.4754 30896785 PMC6438425

[15] Drier Y., Cotton M.J., Williamson K.E., Gillespie S.M., Ryan R.J., Kluk M.J. et al. (2016) An oncogenic MYB feedback loop drives alternate cell fates in adenoid cystic carcinoma. Nat. Genet. 48, 265–272 10.1038/ng.3502 26829750 PMC4767593

[16] Srivastava S.K., Bhardwaj A., Arora S., Singh S., Azim S., Tyagi N. et al. (2015) MYB is a novel regulator of pancreatic tumour growth and metastasis. Br. J. Cancer 113, 1694–1703 10.1038/bjc.2015.400 26657649 PMC4701995

[17] Gautam S., Fioravanti J., Zhu W., Le Gall J.B., Brohawn P., Lacey N.E. et al. (2019) The transcription factor c-Myb regulates CD8(+) T cell stemness and antitumor immunity. Nat. Immunol. 20, 337–349 10.1038/s41590-018-0311-z 30778251 PMC6489499

[18] Cicirò Y. and Sala A. (2021) MYB oncoproteins: emerging players and potential therapeutic targets in human cancer. Oncogenesis 10, 19 10.1038/s41389-021-00309-y 33637673 PMC7910556

[19] Thorsson V., Gibbs D.L., Brown S.D., Wolf D., Bortone D.S., Ou Yang T.H. et al. (2018) The Immune Landscape of Cancer. Immunity 48, 812.e14–830.e14 10.1016/j.immuni.2018.03.023 29628290 PMC5982584

[20] Zeng D., Ye Z., Shen R., Yu G., Wu J., Xiong Y. et al. (2021) IOBR: Multi-omics immuno-oncology biological research to decode tumor microenvironment and signatures. Front. Immunol. 12, 687975 10.3389/fimmu.2021.687975 34276676 PMC8283787

[21] Yoshihara K., Shahmoradgoli M., Martínez E., Vegesna R., Kim H., Torres-Garcia W. et al. (2013) Inferring tumour purity and stromal and immune cell admixture from expression data. Nat. Commun. 4, 2612 10.1038/ncomms3612 24113773 PMC3826632

[22] Ru B., Wong C.N., Tong Y., Zhong J.Y., Zhong S.S.W., Wu W.C. et al. (2019) TISIDB: an integrated repository portal for tumor-immune system interactions. Bioinformatics 35, 4200–4202 10.1093/bioinformatics/btz210 30903160

[23] Li Y., Jin K., van Pelt G.W., van Dam H., Yu X., Mesker W.E. et al. (2016) c-Myb Enhances Breast Cancer Invasion and Metastasis through the Wnt/β-Catenin/Axin2 Pathway. Cancer Res. 76, 3364–3375 10.1158/0008-5472.CAN-15-2302 27197202

[24] Qu X., Yan X., Kong C., Zhu Y., Li H., Pan D. et al. (2019) c-Myb promotes growth and metastasis of colorectal cancer through c-fos-induced epithelial-mesenchymal transition. Cancer Sci. 110, 3183–3196 10.1111/cas.14141 31338937 PMC6778643

[25] Wang W., Yang Y., Chen X., Shao S., Hu S. and Zhang T. (2019) MAGI1 mediates tumor metastasis through c-Myb/miR-520h/MAGI1 signaling pathway in renal cell carcinoma. Apoptosis: Int. J. Program. Cell Death 24, 837–848 10.1007/s10495-019-01562-8 31352641

[26] Zhao X., Zhang W. and Ji W. (2018) YB-1 promotes laryngeal squamous cell carcinoma progression by inducing miR-155 expression via c-Myb. Fut. Oncol. (London, England) 14, 1579–1589 10.2217/fon-2018-0058 29517281

[27] Engelhard V., Conejo-Garcia J.R., Ahmed R., Nelson B.H., Willard-Gallo K., Bruno T.C. et al. (2021) B cells and cancer. Cancer Cell. 39, 1293–1296 10.1016/j.ccell.2021.09.007 34597591

[28] Borst J., Ahrends T., Bąbała N., Melief C.J.M. and Kastenmüller W. (2018) CD4(+) T cell help in cancer immunology and immunotherapy. Nat. Rev. Immunol. 18, 635–647 10.1038/s41577-018-0044-0 30057419

[29] Lauer S. and Gresham D. (2019) An evolving view of copy number variants. Curr. Genet. 65, 1287–1295 10.1007/s00294-019-00980-0 31076843

[30] Goldman M.J., Craft B., Hastie M., Repečka K., McDade F., Kamath A. et al. (2020) Visualizing and interpreting cancer genomics data via the Xena platform. Nat. Biotechnol. 38, 675–678 10.1038/s41587-020-0546-8 32444850 PMC7386072

[31] Chandrashekar D.S., Karthikeyan S.K., Korla P.K., Patel H., Shovon A.R., Athar M. et al. (2022) UALCAN: An update to the integrated cancer data analysis platform. Neoplasia 25, 18–27 10.1016/j.neo.2022.01.001 35078134 PMC8788199

[32] Blum A., Wang P. and Zenklusen J.C. (2018) SnapShot: TCGA-analyzed tumors. Cell 173, 530 10.1016/j.cell.2018.03.059 29625059

[33] Carithers L.J. and Moore H.M. (2015) The Genotype-Tissue Expression (GTEx) Project. Biopreserv. Biobanking 13, 307–308 10.1089/bio.2015.29031.hmm PMC4692118 26484569

[34] Omasits U., Ahrens C.H., Müller S. and Wollscheid B. (2014) Protter: interactive protein feature visualization and integration with experimental proteomic data. Bioinformatics 30, 884–886 10.1093/bioinformatics/btt607 24162465

[35] Szklarczyk D., Gable A.L., Nastou K.C., Lyon D., Kirsch R., Pyysalo S. et al. (2021) The STRING database in 2021: customizable protein-protein networks, and functional characterization of user-uploaded gene/measurement sets. Nucleic Acids Res. 49, D605–D612 10.1093/nar/gkaa1074 33237311 PMC7779004

[36] Li T., Fu J., Zeng Z., Cohen D., Li J., Chen Q. et al. (2020) TIMER2.0 for analysis of tumor-infiltrating immune cells. Nucleic Acids Res. 48, W509–W514 10.1093/nar/gkaa407 32442275 PMC7319575

[37] Wu C., Jin X., Tsueng G., Afrasiabi C. and Su A.I. (2016) BioGPS: building your own mash-up of gene annotations and expression profiles. Nucleic Acids Res. 44, D313–D316 10.1093/nar/gkv1104 26578587 PMC4702805

[38] Fu J., Li K., Zhang W., Wan C., Zhang J., Jiang P. et al. (2020) Large-scale public data reuse to model immunotherapy response and resistance. Genome Medicine 12, 21 10.1186/s13073-020-0721-z 32102694 PMC7045518

[39] Cerami E., Gao J., Dogrusoz U., Gross B.E., Sumer S.O., Aksoy B.A. et al. (2012) The cBio cancer genomics portal: an open platform for exploring multidimensional cancer genomics data. Cancer Discov. 2, 401–404 10.1158/2159-8290.CD-12-0095 22588877 PMC3956037

[40] Gao J., Aksoy B.A., Dogrusoz U., Dresdner G., Gross B., Sumer S.O. et al. (2013) Integrative analysis of complex cancer genomics and clinical profiles using the cBioPortal. Science Signaling 6, pl1 10.1126/scisignal.2004088 23550210 PMC4160307

