# Supplementary material for: The prognostic and immunological role of MYB: from bladder cancer validation to pan-cancer analysis
Source: Biosci Rep. 2023 Apr 6;43(4):BSR20222627. doi: 10.1042/BSR20222627 (PMC10086116; doi:10.1042/BSR20222627)
Supplement: Supplementary Figure S1 [file BSR-2022-2627_supp.pdf]

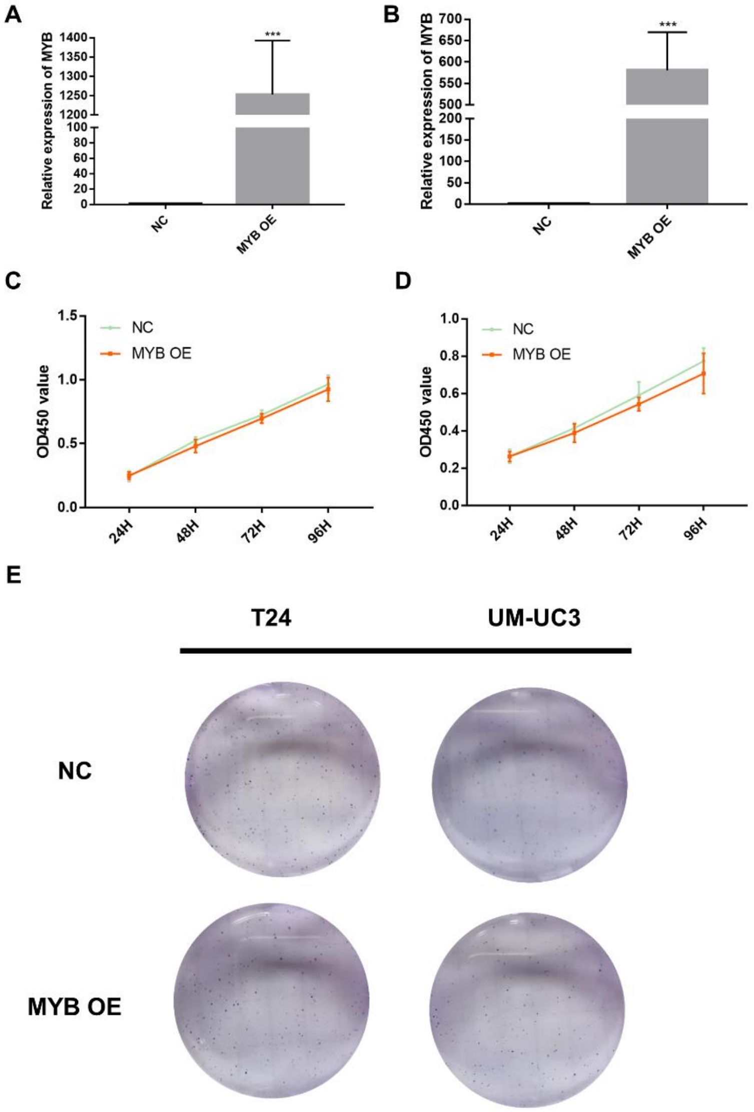


Supplementary Figure 1: MYB has no effect on bladder cancer cell proliferation. (A) The overexpression efficiency of MYB in T24 cells was identified by qRT-PCR. (B) The overexpression efficiency of MYB in UM-UC3 cells was identified by qRT-PCR. (C) The CCK-8 experiment confirmed that overexpression of MYB had no effect on the proliferation of T24 cells. (D) The CCK-8 experiment confirmed that overexpression of MYB had no effect on the proliferation of UM-UC3 cells. (E) The colony formation assay confirmed that overexpression of MYB had no effect on the proliferation of T24 and UM-UC3 cells.
